# From Persuasion to Partnership: Evaluating the Practicalities, Ethics, and Evidence for Implementing Motivational Interviewing in Veterinary Practice

**DOI:** 10.3390/ani16131972

**Published:** 2026-06-26

**Authors:** M. Carolyn Gates, Clare J. Phythian, Eileen Britt

**Affiliations:** 1 AkoVet Limited, Palmerston North 4410, New Zealand; 2 School of Psychology, Speech, and Hearing, University of Canterbury, Christchurch 8140, New Zealand; eileen.britt@canterbury.ac.nz

**Keywords:** motivational interviewing, veterinary communication, behaviour change, client engagement, animal welfare, decision-making, ethics

## Abstract

Veterinary medicine fundamentally revolves around working with people to positively influence how they care for their animals. Veterinarians have traditionally used directive advice-giving when providing clients with recommendations, which can inadvertently push clients further away from change despite good intentions by evoking resistance and leaving the underlying motivational and contextual barriers to change unaddressed. Motivational interviewing (MI), a collaborative communication approach originally developed for addiction counselling, has been widely adapted across many fields because of its proven effectiveness in strengthening intrinsic motivation to change. MI was first applied in veterinary medicine approximately a decade ago with a small but growing evidence base. This review introduces the theoretical foundations of MI, how it can be applied within different types of clinical consultations, as well as the challenges of developing and sustaining competency in practice and ethical implications specific to the veterinarian–client–animal relationship, including the proxy motivation problem where clients bear the costs of behaviour change for benefits experienced primarily by their animal. This review then critically appraises the veterinary MI literature, which has largely focused on communication training outcomes with limited research on the downstream effects on client behaviour or animal welfare, highlighting important research gaps to promote an increased uptake of MI in clinical practice.

## Simple Summary

Veterinarians depend on clients making behaviour changes to improve animal welfare, yet the profession has traditionally relied on directive advice-giving that can generate resistance rather than motivation. Motivational interviewing is a collaborative communication approach, originally developed for addiction counselling, that helps people find their own reasons for change and build the motivation needed to act on clinical recommendations. It has been widely used across human healthcare because of its proven effectiveness and is now being applied in veterinary medicine. This review examines what motivational interviewing is, how it can be used in different types of veterinary consultations, and the practical and ethical challenges unique to a profession where clients are making behaviour changes for the benefit of an animal rather than themselves. A critical look at the veterinary research finds that while early results are encouraging, most studies have focused on whether training changes how veterinarians communicate rather than whether those changes actually lead to better outcomes for animals. This review identifies the most important gaps in the current evidence and lays the groundwork for more rigorous research into motivational interviewing as a practical tool for improving animal care.

## 1. Introduction

Veterinary medicine is a profession that fundamentally revolves around working with people to prevent, diagnose, and treat disease in animals. While the last few decades have seen significant advances in the available diagnostic tests, medications, and procedures [1], the profession has been much slower in translating the recommendations provided during consultations into behaviour change that improves animal welfare, as evidenced by the high rates of preventable diseases [2,3,4,5,6] and low rates of adherence to veterinary advice that have been reported across many different species [7,8]. Traditionally, poor adherence has been attributed to client-side factors such as financial constraints, lifestyle limitations, competing priorities, differing perceptions of risk, and ambivalence about the costs and benefits of different management options. However, there is also growing recognition that how veterinarians communicate with clients to give advice and not just what they advise plays an important role in whether change actually happens [9,10,11]. Simply providing clearer or more detailed information does not reliably resolve barriers to change because these are often motivational and contextual rather than stemming from a lack of client awareness or knowledge [12,13].

Research into veterinary communication has consistently found that veterinarians have a predominantly directive and paternalistic style, where they typically set the agenda, do most of the talking, rely heavily on closed questions, and focus on information delivery rather than exploring what the client thinks, values, or wants [14,15,16,17]. Despite good intentions, this approach may actively undermine the outcomes it seeks to produce. Directive, persuasive communication is more likely to generate psychological reactance than to build the internal motivation that produces sustained behaviour change, whereas clients are more likely to act on advice when they feel their perspective has been heard, their autonomy respected, and their own reasons for change acknowledged [13,18,19]. Shared decision-making has been advocated as a more effective model for clinical communication in both human and veterinary healthcare, but even where it has been widely endorsed, practitioners struggle with its implementation in practice, stemming from both a lack of appropriate communications skills training and the tendency to revert to old communication patterns under the time pressure of routine consultations [19,20].

Motivational interviewing (MI) is a collaborative, goal-oriented approach to having conversations that focus on helping people explore their own reasons for change, resolve ambivalence, and develop an appropriate plan for taking action [21]. Developed initially for addiction counselling and subsequently adapted across a wide range of settings, including healthcare [22], MI has attracted growing attention in veterinary medicine as an evidence-based framework for addressing the communication challenges described above [23,24]. However, its application to veterinary practice has received limited critical examination, and veterinary consultations present structural and ethical challenges that distinguish them from the human healthcare settings where MI was developed and most extensively studied. The most significant of these is the veterinarian–client–animal relationship, where professional duties extend beyond the person in the room to an animal who cannot participate in the conversation, and where the person being asked to change their behaviour is most often not the one who will directly experience the benefits of that change.

This narrative review examines what MI is and how it works, how its application can be shaped within the structural features of veterinary consultations, the challenges of training and sustaining MI competency in clinical practice, and the ethical dimensions of MI specific to veterinary contexts. This paper then concludes with a critical review of the current veterinary evidence base and identifies research priorities to support the responsible and sustainable integration of MI into veterinary practice. This review draws on all known veterinary MI studies, which remain relatively few since Scrase and Reyher first described its application to veterinary medicine in 2015 [25]. These are considered alongside selected examples from the human healthcare literature, where they provide useful theoretical grounding or context.

## 2. Methods

This paper is a narrative review of the theoretical foundations, clinical applications, training evidence, ethical dimensions, and current veterinary evidence base for motivational interviewing in veterinary practice, supplemented by a systematic search of the veterinary MI literature.

The veterinary MI evidence base was identified through a systematic search of PubMed, Web of Science, and CAB abstracts using the search terms ‘motivational interviewing,’ ‘behaviour change counselling,’ ‘client communication,’ and ‘veterinary’ or ‘animal health,’ used individually and in combination. Forward and backward citation searching was conducted from identified papers to ensure completeness. Studies were included if they were conducted in a veterinary or animal health context and involved motivational interviewing as an intervention, training approach, or subject of direct investigation. There were no restrictions on study design, species, or publication date. Review articles, opinion pieces, and papers discussing MI in passing without it being a primary focus of the study were excluded.

The broader narrative review drew on the human healthcare MI literature to provide theoretical grounding and contextual comparison, with papers identified through database searches and hand-searching of reference lists of key MI texts and reviews. Given the narrative rather than systematic nature of this component, no formal inclusion or exclusion criteria were applied, and sources were selected on the basis of relevance to the topics under review.

Where existing frameworks from human healthcare did not adequately address the structural features of veterinary practice, new conceptual frameworks were developed by the authors drawing on their combined experience in veterinary medicine, motivational interviewing research, and behaviour change communication. These frameworks are presented as original contributions intended to guide both clinical practice and future research.

## 3. Motivational Interviewing: What It Is and How It Works

At its core, MI is a way of being present with another person in a conversation that helps them work through ambivalence and find their own pathway to making positive behavioural changes. While many of the foundational skills in MI overlap with those taught in other communication approaches, what distinguishes MI is the mindset that practitioners bring to the conversation and how those skills are directed towards evoking the client’s own motivations for change and supporting them to develop actionable plans. Throughout this paper, the term practitioner is used to refer to the person delivering MI, whether a veterinarian, veterinary nurse, or other member of the clinical team, since the principles and skills described apply across these roles.

**Figure 1:**
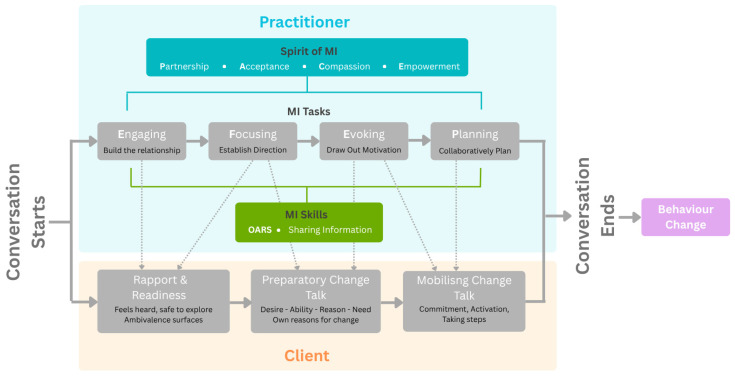
The motivational interviewing (MI) framework illustrating practitioner contributions and client progression from ambivalence to action. OARS refers to the core communication skills of open-ended questions, affirmations, reflections, and summaries. Solid arrows indicate the sequential progression through MI tasks (practitioner) and client language stages (client). Dashed arrows indicate how practitioner MI skills influence client progression through the conversation.

As illustrated in Figure 1, MI can be understood as a two-actor framework. The practitioner (1) brings the spirit of MI as the overarching principle in how they regard and relate to the client, (2) uses the four tasks of MI to guide the conversation toward a shared goal strategically, and (3) applies core MI micro-skills, including open-ended questions, affirmations, reflections, and summaries (collectively referred to as OARS), as well as the Ask-Provide-Ask framework for sharing information, throughout the conversation to support the spirit and tasks in practice. The client, in response to the practitioner’s facilitation, moves from building rapport with the practitioner and becoming ready to discuss change, through preparatory change talk and toward mobilising language and commitment to actions that ideally produce behavioural change outside the session. The following subsections describe each of these components in turn.

### 3.1. The Spirit of MI

Miller and Rollnick [21] describe the spirit of MI as the foundational attitude that underpins the use of MI in practice and divides it into four interrelated elements: Partnership, Acceptance, Compassion, and Empowerment. Partnership means that the practitioner works with the client rather than on the client, treating the consultation as a collaborative interaction between two people with different but complementary knowledge. Acceptance encompasses showing a non-judgmental attitude, taking an interest in the client’s prior experiences, and understanding their unique perspective. Compassion reflects a commitment to prioritising the client’s best interests rather than the practitioner’s preferred outcome. Empowerment recognises that motivation for change cannot be imposed by an external source but must be evoked from within the client by drawing on their own values, strengths, and resources. Without the spirit of MI as its foundation, the use of MI tasks and skills risks becoming a form of persuasion or manipulation rather than true collaboration.

### 3.2. The Four Tasks of MI

MI is structured around four sequential but iterative tasks that guide the conversation towards change: Engaging, Focusing, Evoking, and Planning [21]. Unlike a directing style, where the practitioner sets the agenda and drives toward solutions, or a following style, where the practitioner responds to whatever the client raises without structure, MI occupies a guiding position that is oriented purposefully and directionally toward the client’s own goals and pace rather than the practitioner’s preferred outcome and timing.

Engaging establishes the relational foundation with the practitioner, building rapport, expressing curiosity about the client’s perspective, and creating an environment in which the client feels heard and understood rather than assessed. Without adequate engagement, the subsequent tasks are unlikely to be effective. Focusing establishes a shared sense of direction, identifying what the conversation is about and what change, if any, is being considered. Evoking is the heart of MI, drawing out the client’s own motivations for change rather than supplying them externally. Planning consolidates commitment and supports the collaborative development of a realistic plan when the client has sufficient motivation and readiness to act. These tasks do not occur in rigid stages, and a skilled MI practitioner moves between them fluidly in response to where the client is in a given moment, returning to engagement when the relationship feels strained, or stepping back from planning when ambivalence re-emerges. When used appropriately together, these four tasks give MI its directional quality.

### 3.3. Core MI Skills

The OARS skills are the primary conversational tools through which the spirit and tasks of MI are expressed in practice [21]. Open questions invite elaboration and signal curiosity about the client’s perspective, in contrast to closed questions that direct conversation toward practitioner-determined endpoints. Affirmations acknowledge the client’s strengths, efforts, and values, supporting self-efficacy without flattery or paternalistic praise. Reflective listening communicates understanding that helps the client hear their own thinking more clearly, including the ambivalence that often sits beneath the surface of a conversation, and can be used to selectively reinforce change talk and guide the conversation to build and strengthen motivation. Summaries gather and organise what has been said, further reinforcing change talk and demonstrating that the practitioner has been listening carefully.

Sharing information and providing advice, which form a substantial part of any veterinary clinical consultation, are delivered in MI through the Ask-Provide-Ask framework. The practitioner first asks what the client already knows or wants to know about the topic and listens to the client’s responses to determine what information is appropriate to provide to supplement the client’s existing knowledge. The practitioner then asks the client for permission to share additional information or their professional opinion on the topic before providing that information in a neutral and accessible way if the client consents. If the client does not consent, this presents an opportunity for the practitioner to explore barriers to engaging with the topic. If information is shared, the practitioner then asks the client what they thought about it. This approach preserves autonomy, recognises that the client comes with their own knowledge, and reinforces the partnership and guiding stance that characterises MI practice [21].

Another core focus of MI is learning to recognise and selectively respond to change talk, which includes the client’s own statements about reasons, desire, ability, needs, or commitment to change, and sustain talk, which includes statements the client makes that favour the status quo. Practitioners are trained to reflect and reinforce change talk while responding to sustain talk with curiosity rather than counterargument, since arguing against sustain talk tends to entrench it. Stronger and more frequent change talk, particularly when it shifts toward commitment language, is associated with subsequent behaviour change [26].

When the conversation becomes strained or the client is perceived as being resistant, MI frames this as a signal about the quality of the interaction rather than a problem with the client to be overcome. Miller and Rollnick [21] distinguished between two forms this can take: sustain talk and discord. Sustain talk reflects the client’s own reasons for maintaining the status quo, while discord reflects a rupture in the relational dynamic between practitioner and client. These can occur separately or together. These situations call for the practitioner to adjust their approach and demonstrate the spirit of MI rather than increasing pressure on the client. Effective responses include simple reflection, shifting focus, emphasising the client’s autonomy, and stepping back to re-engage before moving forward again [21,27].

Avoiding behaviours that undermine behaviour change conversations is as important as learning the skills that support them. The fixing reflex, also known as the righting reflex, is the instinct to correct, advise, and persuade that comes naturally to practitioners trained to identify problems and provide solutions. However, in the context of behaviour change, it tends to further entrench ambivalence rather than resolve it. MI-nonadherent behaviours, which include disagreeing, arguing, correcting, shaming, criticising, and providing unsolicited advice, are all expressions of the fixing reflex that have been negatively correlated with behaviour change outcomes [21]. MI practice requires learning to recognise and resist these patterns by listening with curiosity and without judgement rather than defaulting to advice and correction.

### 3.4. How MI Works: Theoretical Mechanisms

Understanding how MI produces behaviour change requires examining both what practitioners do and why it works. At its core, MI is built on the premise that people are more likely to change when they verbally articulate their own reasons for change and develop a plan of action that fits within their own life circumstances rather than being directed towards a solution by the practitioner. Two complementary hypotheses have been proposed to explain why MI produces behavioural changes: the technical hypothesis and the relational hypothesis [26,28]. The technical hypothesis proposes that MI works through a specific linguistic mechanism: MI-consistent practitioner behaviour evokes change talk from the client, change talk strengthens commitment language, and commitment language predicts subsequent behaviour change. In other words, the client believes what they hear themselves speak. The relational hypothesis proposes that the therapeutic alliance created by MI’s empathic and autonomy-supportive style increases client engagement and openness to change, independently of specific linguistic events. Evidence from human healthcare supports that both mechanisms are important, although their relative contribution may vary across different contexts [26].

The theoretical foundations of MI are directly connected to Self-Determination Theory (SDT) [29], which proposes that sustained behaviour change depends on satisfying three basic psychological needs: autonomy, the sense that one’s actions are self-chosen and consistent with one’s values; competence, the sense of being capable of carrying out the behaviour effectively; and relatedness, the sense of feeling understood and connected to others. While extrinsic motivation may produce short-term compliance, it rarely sustains behaviour change once those external pressures or obligations are removed. MI’s emphasis on evoking intrinsic motivation and supporting autonomy maps directly onto SDT’s account of what conditions need to be in place for change to be both initiated and maintained.

This connection helps explain why the spirit of MI is so closely linked to its effectiveness. A practitioner who applies MI skills without building a relationship that supports client autonomy is unlikely to meet the client’s psychological needs that SDT identifies as necessary for sustained change.

### 3.5. What Counts as MI: The Fidelity Question

Determining whether a practitioner is actually delivering MI requires a systematic assessment of recorded consultations against validated criteria. Fidelity to MI is most commonly assessed using the Motivational Interviewing Treatment Integrity code (MITI) [30], the latest version of which is the MITI 4.2.1 [31], which evaluates practitioner behaviour across two domains: relational and technical. While other fidelity instruments exist, including the Behaviour Change Counselling Index (BECCI) [32] and the Motivational Interviewing Skills Code (MISC) [33], the MITI has become the most widely used standard in both research and training contexts. The relational domain captures global qualities of the interaction, including the degree to which the practitioner demonstrates empathy and partnership, while the technical domain captures the degree to which the practitioner cultivates change talk and softens sustain talk. The MITI 4.2.1 also includes specific behavioural counts of various practitioner behaviours, including the use of questions, reflections, and affirmations, seeking collaboration, and emphasising autonomy. Together, these produce scores for practitioner performance that can be compared against thresholds for fair or good proficiency. However, it should be noted that these thresholds were developed through expert consensus rather than empirical research, and several studies have found patterns of client change talk and behaviour consistent with MI predictions even when practitioners do not meet them, raising questions about their validity as a universal benchmark [34,35]. Furthermore, the MITI 4.2.1 requires a minimum 20 min conversation sample to generate reliable scores, which presents a practical challenge in medical and veterinary consultations where interactions are often considerably shorter.

## 4. Motivational Interviewing in Veterinary Consultations: Clinical Contexts, Applications, and Constraints

Motivational interviewing was originally developed for addiction counselling, and its subsequent adaptation to other contexts, including brief medical consultations, has required pragmatic trade-offs between fidelity to the full MI framework and what is achievable in time-limited clinical settings [22]. Veterinary medicine presents even further structural and ethical challenges that distinguish it from the human healthcare contexts where MI was developed. This section examines the clinical contexts in which MI is relevant in veterinary practice, proposes a three-level framework for calibrating the approach to different consultation types, and analyses the features of veterinary consultations that make behaviour change support distinctively challenging, including the barriers that commonly prevent change and the diagnostic frameworks that help identify them.

### 4.1. Clinical Contexts and Applications

Veterinary practice encompasses a diverse range of clinical settings and consultation types that vary in their complexity, duration, and the depth of MI engagement they require to achieve behaviour change. While the nature of what needs to change and who needs to change it differs considerably across companion animal, production animal, and non-clinical contexts, the underlying communication challenge of helping people find their own reasons to act remains consistent.

In companion animal practice, the most common use cases for MI are likely to be with conditions that cannot be effectively managed without significant and ongoing changes to owner behaviour, including obesity, dental disease, behavioural problems, and chronic diseases such as diabetes and osteoarthritis. These may require owners to make changes to their feeding habits, home care routines, medication management, and the way they interact with their animals on a daily basis. Many of these changes can be difficult to establish and even harder to maintain over time. Client-centred communication has been shown to improve owner adherence in these contexts [36] and MI offers a structured framework for putting that into practice.

Behaviour change in production animal practice is often at the farm systems level, where there may be significant financial and practical costs to shifting established management practices for herd health, antimicrobial stewardship, and biosecurity. The most extensive MI research in veterinary contexts has been conducted for dairy herd health management, particularly around mastitis and lameness [15,37], and the potential role of MI in biosecurity training has been proposed, although formal evaluations have not yet been conducted [38]. The costs and benefits of change in these contexts are more readily framed in economic terms than in companion animal practice, and MI may be particularly appropriate where ambivalence about the costs of change intersects with motivation to improve herd welfare and productivity.

MI may also be valuable in consultations where the goal is not long-term behaviour change but supporting a client through a difficult decision, as in end-of-life care. Reflective listening, summarising, and supporting autonomy can help clients reach decisions consistent with their own values and their animal’s needs even under conditions of grief and uncertainty [39]. The same skills that support collaborative client conversations are applicable to team leadership, mentoring, workplace conflict, and supporting colleagues who are struggling. Veterinarians participating in MI training have spontaneously identified its relevance to collegial relationships as well as client consultations [40], and evidence from healthcare settings suggests MI training is associated with improved clinician wellbeing, consultation satisfaction, and reduced moral distress [41,42], which supports the case for MI as a broader professional practice framework rather than solely a client communication tool.

### 4.2. Calibrating MI to the Consultation: A Levelled Framework

Motivational interviewing was originally developed for addiction counselling, where it is common to have longer sessions that focus primarily on behaviour change. In medical and veterinary settings, there is a wide range of case presentations, and not every clinical consultation requires the full MI framework. Rollnick and colleagues [43] first articulated this challenge in proposing brief MI for medical settings, and the subsequent development of behaviour change counselling built on this by distinguishing between brief advice, MI adapted for time-limited consultations, and full MI [22,44]. Research on brief interventions has since shown that MI-consistent communication can produce meaningful effects across a range of health behaviours even when delivered in a compressed form [45,46].

Veterinary practice requires a similar framework for matching the communication approach to what a given consultation actually demands for client behaviour change. The following three-level framework draws on existing distinctions in the MI literature to provide a practical clinical decision-making tool that is proposed as a way of thinking about which communication approach best serves the animal, the client, and the purpose of the consultation.

Level 1 (Minimal MI) consultations are straightforward encounters where the diagnosis is clear, the recommended actions are simple, and the client is willing and able to carry them out. This can include situations such as vaccinating a dog that will be kennelled, performing surgery to repair a laceration on a horse’s leg, or advising a sheep farmer on the best deworming product to use. Directive communication is often appropriate and efficient here. However, MI conversational skills, particularly open-ended questions and reflective listening, remain valuable for building rapport and ensuring the client has understood. The full MI process for evoking reasons for change may not be necessary or indicated, but practitioners should remain alert to unexpected ambivalence, since a client who expresses hesitation about completing a course of medication or uncertainty about a recommended procedure may warrant a shift toward Level 2.

Level 2 (Adapted MI) consultations involve some degree of client ambivalence or uncertainty, whether about a clinical decision with multiple options or about their readiness to commit to a recommended change. Common clinical situations where this occurs include preventive care discussions, medication adherence conversations, minor management adjustments, and presentations with several diagnostic or treatment pathways to choose between. The clinical focus is usually clear, so the bulk of the work involves exploring the client’s perspective, understanding what is driving their uncertainty, and helping them work through ambivalence to reach a decision from the available options. OARS skills and the Ask-Offer-Ask framework are the primary tools, with the goal of a collaborative rather than directive interaction. This corresponds broadly to what the human healthcare literature describes as behaviour change counselling [45,46].

Level 3 (Full MI) consultations involve significant ambivalence, complex behaviour change demands, competing values, entrenched patterns, or decisions with substantial welfare implications. Chronic disease management, major changes to farm management practices, repeated non-adherence, and situations where the client’s stated preferences conflict with the animal’s welfare needs all potentially fall into this category. This is where the full MI framework is most valuable, with deliberate attention to change talk and sustain talk, sustained exploration of the client’s own motivations, and extended engagement that gives ambivalence room to shift. These consultations may require more time than a standard clinical appointment allows, and in some cases, the behaviour change conversation is better spread across multiple interactions.

Level selection is not a fixed characteristic of a consultation type but rather a clinical judgement made in real time based on what the client presents. For example, a simple appointment for an ear infection in a dog easily becomes a Level 3 conversation if the owner is reluctant to implement preventive care measures. Equally, a herd health visit expected to require extended MI engagement may resolve quickly if the farmer has already made a clear decision. The levels are intended to provide orientation without being prescriptive.

### 4.3. The Triadic Relationship and Shared Moral Agency

A fundamental structural difference with veterinary consultations is that they involve three parties by default: the veterinarian, the client, and the animal. While situations where one party makes decisions on behalf of another do arise in human healthcare, such as in paediatric medicine or cases involving cognitive impairment, in veterinary medicine, two parties routinely share agency over decisions that affect a third party who cannot participate in the conversation, articulate their preferences, or consent to what is proposed. In both human and veterinary contexts, legal and regulatory frameworks exist to protect the interests of the party who cannot advocate for themselves, and a practitioner’s professional obligations to their patient can override client or parental preferences where welfare is seriously compromised. In veterinary medicine, this framework is established through the Veterinarian–Client–Patient Relationship (VCPR) and through animal welfare legislation, though the balance between client autonomy and animal welfare obligations is not always clearly resolved and requires ongoing professional judgement. The legal weighting of these obligations varies across jurisdictions, and the framework presented here reflects common law jurisdictions such as New Zealand, the United Kingdom, and Australia, where animal welfare statutes place obligations on veterinarians that exist independently of the client relationship. It is also worth noting that even where the veterinarian and client are formally equal parties to a service contract, the practical reality of veterinary consultations typically involves a significant asymmetry of clinical knowledge and authority between the two. Addressing this asymmetry, so that it does not prevent the client from participating meaningfully in decisions about their animal’s care, is itself one of the central reasons MI is relevant to veterinary practice.

Rather than treating this triadic structure purely as a source of tension, it can be reframed as an opportunity for a different type of collaborative practice with the veterinarian and client functioning as a collective moral agent on behalf of the animal who cannot advocate for itself, each contributing different but complementary knowledge, with the veterinarian bringing clinical expertise about the animal’s medical and behavioural status, and the client bringing contextual understanding of the animal’s life, personality, and the practical realities of the household or farm. This concept of shared moral agency is distinct from diffusion of responsibility, where the presence of multiple parties reduces individual accountability. Shared moral agency instead calls for greater engagement from both parties, precisely because the animal cannot advocate for itself, drawing on broader thinking about collective responsibility in ethics [47,48] and on the argument that veterinarians have a professional obligation to act as strong advocates for their animal patients [49]. MI is particularly well suited to facilitating this type of shared agency because its spirit of partnership, acceptance, and authentic curiosity about the client’s perspective creates the conditions in which meaningful collaboration becomes possible.

### 4.4. Understanding Behaviour Change Barriers in Veterinary Contexts

A recurring challenge in veterinary practice is that the person being asked to change their behaviour is not the one who will most directly benefit from that change, and the intrinsic motivation that MI is designed to evoke cannot be assumed to exist in the same form it might in other clinical contexts. The relationship between a client and their animal also shapes the motivational landscape considerably. A dog owner who regards their animal as a family member may be highly motivated to act but constrained by finances, time, or competing household priorities [50]. A production animal farmer, by contrast, may frame animal welfare decisions primarily through the lens of economic viability, herd productivity, or practical feasibility, and recommendations that do not engage with that frame are unlikely to produce change regardless of how well they are delivered [51]. Understanding what type of relationship the client has with their animal and what values and constraints shape their decision-making is, therefore, a necessary precondition for effective MI engagement. Clients who appear disengaged or non-compliant may be dealing with barriers that are not immediately visible in the consultation, and taking time to identify what is actually limiting change is often more productive than assuming ambivalence and responding with MI by default.

The COM-B model [52] provides a useful diagnostic framework for this purpose. The model identifies three conditions required for behaviour change: capability (the physical and psychological ability to perform the behaviour), opportunity (the environmental and social conditions that make it possible), and motivation (the drives and reasoning that direct action). Identifying which of these is lacking helps determine what type of intervention is most likely to be useful. In many veterinary behaviour change contexts, the barrier is not capability. A client who is not managing their dog’s weight or administering daily medication is unlikely to lack the knowledge or physical ability to do so. Opportunity barriers are more common than they might first appear, with financial constraints, time pressures, household dynamics, and competing priorities all limiting what is practically achievable, regardless of how motivated a client might be. Where these barriers are present, MI’s role is not to evoke motivation for a change that is out of reach, but to explore what is actually possible within the client’s real circumstances. The barrier is most often motivational, however, and specifically, a deficit in intrinsic motivation because the behaviour demands ongoing effort for benefits experienced by another being entirely. Defaulting to providing the client with more information in these situations leaves the motivational barrier untouched, which is why advice-giving so frequently fails to produce sustained change.

The Transtheoretical Model [53] adds a temporal dimension, recognising that behaviour change unfolds across stages rather than as a single event. These range from precontemplation, where the person does not yet see the need to change, through contemplation and preparation, to action and longer-term maintenance, with the possibility of relapse at any point. A client who is not acting on a recommended change may be at any of these stages, from not yet recognising the problem as relevant to their situation, through weighing up whether change is worth the effort, to actively attempting to sustain a change they have already started. A farmer who does not perceive lame cows as suffering occupies a different position from one who recognises the problem but lacks the resources to address it, and each requires a different approach. Knowing where a client sits in this process shapes how MI is used and what it can reasonably be expected to achieve.

The key clinical implication is that MI in veterinary contexts requires more deliberate attention to the client’s own values and the relationship between those values and their animal’s welfare than is typically required in human healthcare. Evoking intrinsic motivation in a proxy context means helping the client connect the recommended change to something that matters to them, whether that is their identity as a responsible animal owner, their satisfaction in seeing their animal thrive, or their professional commitment to animal welfare.

## 5. From Training to Sustaining: Developing Motivational Interviewing Competency

Evidence from multiple human healthcare fields suggests that MI can positively influence clinical outcomes and professional wellbeing when it is delivered with sufficient fidelity [54]. As such, there has been growing interest in understanding how MI can be effectively taught, assessed, and maintained in clinical practice settings. MI is a complex communication skill that requires sustained practice with structured feedback and ongoing reflection to help develop and maintain competency over time [55]. Research consistently shows that receiving MI training does not reliably translate into delivering MI with adequate fidelity in practice, and understanding why that gap exists has direct implications for how training programmes are designed and evaluated [56]. This section examines the current state of MI training in healthcare education, the evidence on how the format, intensity, and duration of training impact fidelity, approaches to assessing MI competency, and the barriers and enablers that shape whether MI skills are sustained in clinical practice with a focus on the implications for veterinary medicine.

### 5.1. Teaching MI: Approaches and Current State in Healthcare Education

Although MI training has been increasingly integrated into medical education curricula over the past two decades, how much exposure students receive and how it is delivered varies considerably across institutions. A systematic review by Kaltman and Tankersley [57] found that medical schools can feasibly implement MI training to support students developing beginner levels of proficiency, and that students respond well to case-based approaches that combine didactic instruction with structured role-play and standardised patient encounters. Practical guidance on how to design and deliver such curricula has been developed for medical educators [58,59]. Despite this progress, a nationally representative survey of internal medicine residents in the United States found that while 67.7% reported receiving some MI training in medical school, only 27.2% received further training during residency, and 23.5% reported receiving no MI training at all [60]. Training formats varied considerably, with most relying on lectures and exercises rather than direct observation of real clinical encounters, which only 38.7% reported experiencing. Critically, most respondents in the survey reported rarely or only sometimes using core MI skills in practice, suggesting that exposure to MI training does not reliably translate into clinical application.

MI training has not yet been widely incorporated into veterinary education curricula. Blaxter and colleagues [61] raised the question of whether MI should be taught within veterinary communication skills training, noting both its potential for improving client engagement in complex behaviour change contexts and the ethical considerations that arise when practitioners are trained to influence client decision-making. From a practical perspective, integration would be relatively straightforward since MI is complementary to the Calgary-Cambridge communication model, which is widely taught at veterinary schools and emphasises basic open questioning, listening, and relationship-building skills [62]. However, the way Calgary-Cambridge is typically taught differs fundamentally from MI. Calgary-Cambridge focuses on consultation structure and information transfer. MI is oriented toward evoking motivation and exploring ambivalence, and the spirit of MI, particularly its acceptance of client autonomy and deliberate resistance to the fixing reflex, represents a significant departure from the expert-led communication culture that veterinary training often reinforces. A recent behaviour change training programme for farm advisors provides a promising model for how MI-informed training can be adapted for agricultural advisory contexts with some parallels to veterinary herd health practice [63].

### 5.2. Training Format, Intensity, and Duration

Developing competency in MI requires substantially more training than many programmes are able to deliver in healthcare settings. The Motivational Interviewing Network of Trainers (MINT) recommends a minimum of 16 h of training to begin learning MI, with the explicit acknowledgement that this is not nearly sufficient to achieve full competency. In practice, many training programmes delivered to healthcare practitioners consist of one to four hours, often in the form of a single workshop. The evidence consistently shows that brief, one-off training produces short-term changes in self-reported confidence and knowledge but rarely produces sustained changes in clinical practice [55,64,65].

A more effective model, supported by evidence from both medical and veterinary contexts, involves an extended training programme combining structured workshops with supervised practice between sessions and regular feedback on recorded consultations using validated fidelity tools [66]. A study that involved training Swedish dairy veterinarians used a six-month programme that combined six workshops totalling 36 h, with ongoing recorded practice and MITI-coded feedback, and even then, only 29% of participants reached moderate MITI competency—and those gains were not maintained without continued practice and support [66,67].

The format of training also matters. A comparison of online and in-person training found that both formats can improve knowledge and self-reported skills, but that in-person training with peer interaction and feedback produced stronger outcomes [68]. Live supervision, involving real-time observation and coaching during clinical encounters, has been associated with improved skill acquisition, greater practitioner empathy, and reduced decline in skills over time. However, it is resource-intensive and difficult to implement at scale. Ongoing supervision has been shown to be an effective method for implementing and sustaining MI skills, with a randomised controlled trial finding that structured supervision improved both practitioner fidelity and client outcomes compared to training alone [69].

Embedding MI training into clinical environments requires more than individual training investment, with organisational factors including leadership support, protected time for practice, and peer networks playing a critical role in whether skills are sustained [70]. Mullin and colleagues [71] also noted the difficulty that primary care physicians have in sustaining new communication habits in busy clinical environments, especially when there are many competing demands on their time. Emerging evidence suggests that Artificial Intelligence (AI)-assisted analysis of consultation transcripts may offer a scalable alternative for providing feedback on MI skills, though current tools have significant limitations, particularly in capturing non-verbal communication and the relational dimensions of consultation quality [72,73,74].

### 5.3. Assessing Competency: Fidelity Tools and the Self-Assessment Gap

Accurate assessment of MI competency is essential both for ensuring that practitioners are delivering MI with sufficient fidelity to produce the intended effects and for identifying where further skills development is needed. As described in Section 3.5, the MITI is the most widely used fidelity assessment tool and has been applied extensively in both human healthcare and veterinary MI research [30,31]. However, the MITI presents a practical challenge in clinical settings: reliable coding requires a consultation of at least twenty minutes focused primarily on behaviour change, which does not reflect the reality of most veterinary or medical consultations. The MITI was designed around a 20 min conversation sample, but applying it to a ten-minute appointment covering multiple concerns, or to a brief exchange about medication adherence within a longer consultation, does not produce a reliable picture of practitioner competency.

One solution is to assess competency using simulated consultations with standardised patients or actors, which can be designed specifically to elicit the MI skills being evaluated. This approach is widely used in medical education [57] and is also common in many veterinary communication skills training programmes [75,76]. The limitation is that performance in simulated encounters does not reliably predict performance in real clinical consultations, where time pressure, clinical complexity, and the emotional dynamics of the relationship with a known client all shape communication behaviour in ways that simulation cannot replicate [71].

For briefer consultations that are more consistent with behaviour change counselling, the Behaviour Change Counselling Index (BECCI) offers a validated alternative focused on the practitioner behaviours most relevant in time-limited encounters [32]. The Client Language Easy Rating (CLEAR) system provides a complementary tool for assessing client verbal responses, categorising language into change talk, sustain talk, and neutral talk [77]. Although these tools are better suited to the brief, embedded behaviour change conversations that characterise most interactions in clinical practice, they capture a narrower range of MI competencies than the full MITI.

A critical finding with direct implications for training design is the consistent gap between practitioners’ self-assessed MI skills and their objectively measured fidelity. In a study of 134 MI practitioners receiving ongoing feedback, Beckman and colleagues found only weak associations between self-reported and independently coded MI skills, and that monthly objective feedback over twelve months did little to close that gap [78]. This suggests that practitioners who believe they are communicating collaboratively and effectively may be systematically wrong about their own practice, and self-report alone cannot serve as a reliable basis for identifying the need for further development or for evaluating training outcomes. This is reinforced by McKenzie and colleagues’ analysis of 60 GP consultations in Scotland, where no physicians met beginner-level MITI proficiency and 18% of consultations included confrontation, an MI-nonadherent behaviour, despite MI having been part of Scottish medical education for over two decades [79]. The gap between training and practice is a structural problem that requires structural solutions, not simply more training.

### 5.4. Barriers to Implementation

The barriers to implementing MI in clinical practice operate at multiple levels, which Sannes [80] classified into three broad categories: (1) provider barriers, including limited knowledge and awareness; (2) habitual communication patterns and concerns about the time required; and (3) client and patient barriers, including limited readiness or engagement, and practice barriers, including organisational culture, time constraints, and limited resources for training and supervision.

At the provider level, a common barrier is a misunderstanding of what MI actually is. Associations with addiction counselling, concerns about the time required to use MI in brief consultations, and anxiety about losing professional authority or appearing to withhold expert advice have all been reported amongst physicians [81,82]. In veterinary contexts, the professional expectation that the veterinarian functions as the expert who provides solutions is particularly deeply embedded and may actively conflict with MI’s emphasis on evoking client-generated motivation. Practitioners can learn to use OARS while continuing to approach conversations in a directive way, producing an interaction that looks like MI but lacks its spirit. Effective training programmes need to develop the underlying mindset alongside the skills.

The implementation literature tends to focus on practitioner and organisational barriers, but client factors play an equally important role in determining whether MI succeeds. Clients who are not ready to engage with behaviour change, who have limited trust in the practitioner relationship, or who bring strong competing priorities to the consultation can limit the effectiveness of even well-delivered MI. In veterinary contexts, the proxy motivation problem compounds this: a client who does not perceive their animal’s condition as a problem requiring action may present with a precontemplation profile that even skilled MI engagement cannot readily shift in a single consultation.

At the practice and professional culture level, time pressure is consistently identified as a barrier to sustained MI use [67]. Practitioners who have invested in developing MI skills report that reverting to more directive communication habits is easier under conditions of stress, tiredness, or time constraint, and that maintaining the effort required for MI engagement is difficult without organisational support and collegial reinforcement. To the authors’ knowledge, there are currently few veterinary-specific MI professional development courses available, meaning practitioners who want to maintain and develop their skills after their primary veterinary education training have limited accessible options for doing so.

Personal fit with MI’s communication style is also important. Svensson and Bergeå [67] found that how well MI matched a practitioner’s natural personality and communication preferences was an important factor in post-training use, with some practitioners experiencing MI as a natural extension of how they already communicated, while others found the approach effortful and difficult to sustain.

### 5.5. Organisational and Systemic Enablers

Sustained MI competency requires more than individual training investment. Langlois and Goudreau [83] studied MI implementation in primary care settings and found that organisational support, including leadership endorsement, protected time for practice and feedback, and peer networks of practitioners using the same approach, was essential for MI to become embedded in routine clinical culture rather than remaining an individual skill used inconsistently. Without these conditions, skills acquired through training tend to decay over time, particularly in high-pressure clinical environments [55,72].

Peer observation and collegial feedback represent a practical and relatively low-cost enabler that is underutilised in both medical and veterinary settings. Structured peer review of recorded consultations, where practitioners observe each other’s work and provide feedback against shared criteria, can provide a low-cost mechanism for maintaining skills and building the type of reflective practice culture that individual training alone cannot produce. Evidence from the Swedish dairy veterinarian studies suggests that collegial engagement with MI, rather than isolated individual practice, was associated with more sustained post-training use [84], and this finding is consistent with broader evidence on the role of peer support in sustaining complex communication skills [69].

Practice-level culture change is a related enabler. MI is more likely to be sustained when whole teams adopt a shared communication philosophy rather than when individual practitioners attempt to use it in isolation within a practice culture that defaults to directive communication. In veterinary practice, where consultation culture is often strongly shaped by senior clinicians and practice owners, leadership modelling of MI-consistent communication may be as important as formal training in determining whether the approach takes hold.

Professional bodies and regulatory frameworks have a role to play in creating systemic incentives for MI training uptake. Where communication competency standards are explicit and assessed, practitioners have stronger incentives to invest in skill development. Accrediting MI training as counting toward required continuing education hours would lower the practical barriers to uptake and signal that communication skills are valued alongside clinical and technical competencies. To date, veterinary professional bodies have been slow to develop explicit communication competency frameworks, and this represents a gap that limits the systemic uptake of approaches like MI.

The cost-effectiveness of MI training investment is an important consideration for practices and professional bodies evaluating whether to commit to sustained MI development programmes. Evidence from paediatric primary care suggests that MI training can be cost-effective relative to usual care when downstream behaviour change and health outcomes are taken into account [85].

## 6. Ethical Tensions and Professional Responsibilities

MI is sometimes presented as an inherently ethical approach to clinical communication on the grounds that it respects client autonomy and avoids coercion [86]. In practice, the ethical picture is more complex since its structure is deliberately directional with the practitioner listening selectively for change talk, reinforcing language that favours the target behaviour, and guiding the conversation towards a particular outcome [21]. The central ethical question in MI is therefore not whether influence occurs, but how consciously it is exercised and toward what ends [86,87]. This section examines five dimensions of ethical accountability with MI use in veterinary medicine: the manipulation concern and informed consent, the limits of collaborative practice when the interests of the animal are not aligned with the interests of the owner, practitioner competence and scope of practice, cultural and socioeconomic dimensions, and the habit of ethical reflexivity that responsible MI practice requires.

### 6.1. Practitioner Bias and Manipulation Risk

One of the core ethical tensions in MI occurs when the practitioner has a preferred outcome that differs from what the client wants, needs, or is able to manage in their given circumstances. For example, a veterinarian may consider a newer targeted therapy the safest and most effective option for managing atopic dermatitis in a dog, while the owner may be unable to afford it and wants to explore cheaper alternatives with a less favourable side effect profile. Compassion directs the veterinarian to act in the best interests of the client and animal they are working with, while also simultaneously respecting the client’s autonomy and right to make their own decisions. In these situations, a practitioner may consciously or unconsciously use reinforcing language that supports their preferred outcome, present treatment options selectively, or frame information in ways that subtly signal approval or disapproval. The conversation may appear collaborative on the surface, while, in practice, steering the client toward an outcome that undermines genuine self-determination.

When MI skills are practised without MI spirit, there is an even greater ethical risk of the consultation becoming a sophisticated form of manipulation [88,89]. In veterinary practice, this may occur when there are financial or professional incentives for the veterinarian to recommend particular management options. Production-based remuneration, where a veterinarian’s income is directly tied to the revenue they generate, and corporate practice models that have explicit revenue targets create structural incentives that may consciously or unconsciously align clinical recommendations with financial gain rather than patient welfare [90,91]. A separate but related risk arises where practitioners lack confidence or experience with certain treatment options and may steer clients toward alternatives they are more familiar with, regardless of whether those options best serve the client or animal. Clinical uncertainty can compound this risk further, with practitioners who are less tolerant of uncertainty being more likely to pursue additional diagnostics or treatments as a means of resolving their own discomfort rather than in direct response to clinical need [92].

The spirit of MI, with its commitments to partnership, acceptance, compassion, and empowerment, is the primary ethical safeguard against these risks. Practitioners should be clear, at least in their own reflective practice, about the direction in which they are guiding a conversation and whether that direction genuinely reflects the client’s own emerging motivations or their own clinical preferences. In situations where the two are different, the spirit of MI requires either following the client’s lead as long as it does not compromise animal welfare or being openly transparent about the mismatch between their own preferences and what the client wants.

### 6.2. The Proxy Motivation Problem: When Client and Animal Interests Are Not Aligned

In veterinary medicine, decisions about an animal’s care are made by the owner and veterinarian on behalf of the animal who cannot participate in them. While similar situations do arise in human healthcare settings, such as paediatric care, where parents make decisions on behalf of a child, or cases involving cognitive impairment, where a legal power of attorney acts on behalf of a patient, in veterinary medicine, this is the default condition rather than the exception. This proxy motivation problem means that the client bears real and immediate costs to their time, finances, and daily routines for benefits that may accrue primarily or entirely for their animal [93]. This dynamic plays out across a wide range of veterinary behaviour change contexts. For example, weight management plans require owners to resist their animal’s food-seeking behaviour, often at an emotional cost to a relationship built partly on the pleasure of feeding. Long-term medication protocols impose ongoing financial and practical burdens. Biosecurity changes on farms may demand significant infrastructure investment or disruption to established routines, with productivity improvements that are probabilistic rather than guaranteed.

Ethical use of MI, in this context, requires explicit acknowledgement of the burden being placed on the client rather than framing their ambivalence as something to overcome. A client who is uncertain about implementing a weight management plan may be making a legitimate and reasonable assessment of competing responsibilities and constraints. The absence of direct personal benefit makes evoking intrinsic motivation harder than in human healthcare contexts, and the practitioner must work more deliberately to help the client connect the recommended change to something that matters to them, whether that is their identity as a responsible animal owner, their relationship with their animal, or their own values around animal welfare. MI has demonstrated effectiveness in paediatric contexts, where adults make decisions on behalf of children who cannot fully participate in the conversation [94,95]. However, whether this translates to veterinary settings is an open question since the relationship between a parent and child is fundamentally different from the relationship between an owner and an animal, both in the nature of the attachment and in the emotional, financial, and practical costs that an owner has to bear.

There are clinical situations where professional obligations to animal welfare take precedence over client autonomy and may require the veterinarian to step outside the MI framework altogether. Emerging thinking about animal autonomy recognises that animals have an interest in experiencing a life with positive mental states, and that this interest deserves explicit consideration in clinical decision-making [96,97]. The Five Domains framework provides a structured basis for assessing animal welfare and identifying where intervention is required [98,99]. Cases of clear and immediate suffering, neglect, cruelty, or situations requiring mandatory reporting are contexts in which the deliberate exploration of ambivalence is neither ethically defensible nor practically appropriate. However, even in these situations, the spirit of MI, particularly its emphasis on respectful engagement and avoiding blame, may still inform how the practitioner communicates with the owner since the goal is not only to address the immediate situation but to support better outcomes for the animal going forward.

### 6.3. Practitioner Competence and Scope of Practice

Practitioner proficiency is an ethical concern in its own right. Because MI is directional, a practitioner who uses MI skills poorly can unintentionally cause harm by placing subtle pressure on a client’s reasoning process without either party being aware of it [89,100]. A double-sided reflection that consistently positions change talk before sustain talk may inadvertently shift a client’s focus in ways that neither party intends. Research consistently shows that MI fidelity is difficult to maintain without ongoing coaching and structured feedback, particularly in busy clinical settings [56,101], and the ethics of MI become harder to defend if practitioners cannot sustain the level of competence required to use it responsibly. This concern is most consequential in end-of-life consultations, where an owner guided toward a euthanasia decision they were not ready to make cannot reverse that decision. The potential harms of low-fidelity MI remain poorly understood in the literature [101] and this gap is particularly significant in contexts where decisions are irreversible.

Veterinary consultations across a range of contexts can trigger significant emotional responses in clients, not only in end-of-life situations but in any consultation where the client’s relationship with their animal, their sense of responsibility, or their financial constraints are implicated. MI’s emphasis on exploring ambivalence and eliciting underlying values may, in skilled hands, open space for meaningful reflection. In less skilled hands, or with a client who is already emotionally overwhelmed, it risks drawing out distress that the veterinarian is neither equipped nor positioned to contain. Veterinarians are not trained counsellors, and the consulting room is not a therapeutic space. The human–animal bond can be a primary attachment relationship for some clients, particularly those who are isolated or bereaved, and the loss of a companion animal can precipitate significant psychological distress in vulnerable individuals [102,103]. While veterinary professionals consistently identify emotional labour as a contributor to burnout and compassion fatigue, particularly around end-of-life care [104], evidence from other healthcare fields suggests that MI training may reduce this burden by giving practitioners a structured framework for managing emotionally charged conversations rather than defaulting to avoidance or unproductive confrontation [105]. However, the ethical use of MI still requires clarity about the boundaries of the practitioner’s role, including knowing when a client’s needs exceed what a veterinary consultation can appropriately provide, and having referral pathways to mental health support, including veterinary social workers, when that threshold is reached [106,107].

### 6.4. Cultural and Socioeconomic Dimensions

MI’s emphasis on respecting autonomy and following the client’s lead makes it arguably better suited to cross-cultural application than more directive communication approaches [21]. At its core, MI works by understanding decisions through the client’s own values and context rather than imposing an external framework, which makes it more adaptable to cultural variations in how decisions are made and who makes them. MI has been successfully implemented across a range of cultural contexts and languages, with some evidence suggesting its effects may be stronger in minority populations than in majority ones [108].

However, cultural awareness remains important. In contexts where deference to professional authority is the expected social norm, or where family or community members play a central role in health decisions, practitioners need to be alert to how these dynamics shape the conversation rather than treating MI’s collaborative framework as culturally neutral [109,110]. In veterinary medicine, religious and cultural beliefs can also directly affect clinical decisions in ways that require careful navigation. For example, a client who has religious beliefs against euthanasia is not expressing ambivalence to be resolved but communicating a value that must be respected and worked with [111].

The financial structure of veterinary care creates an additional ethical consideration for MI practitioners. Veterinary care is not publicly funded, and what a client can afford directly shapes what options are available to them. A client who declines a recommended treatment because of cost has not expressed ambivalence in the MI sense, but rather, they have made a practical decision within real constraints. Treating financial limitation as a motivational barrier to be explored and resolved risks being both inaccurate and disrespectful. Contextualised care, which recognises that there are often a range of acceptable options for treating or managing a condition and emphasises tailoring recommendations to match the client and animal circumstances, is more consistent with MI spirit than defaulting to gold standard or higher-cost options without acknowledging what is realistically achievable for the client [112,113,114].

### 6.5. Ethical Reflexivity in Practice

There is no simple set of rules for when to use MI and when not to for any given consultation. Practitioners must instead develop an ongoing habit of self-examination that keeps ethical accountability present throughout the interaction rather than treating it as something to be established at the outset and then set aside. Setchell and Dalziel describe this as critical reflexivity, the practice of examining the assumptions, values, and power dynamics that underpin clinical decisions, including the unintended effects of well-intentioned clinical practice [115,116]. Applied to MI, this means making decisions about whether MI is appropriate for the consultation, maintaining honest awareness of whose interests are being served at each point in a conversation, whether the collaborative framework is truly serving the client or obscuring a professional obligation, and whether the practitioner is working within the limits of their competence.

There are three key questions that practitioners can ask themselves to make ethical reflexivity a habit:

- Whose interests am I serving right now: the client’s, the animal’s, and/or my own preferred outcome?
- Am I working within the limits of my competence, and do I know where those limits are in this conversation?
- Is the collaborative framework I am using serving this client, or is it obscuring a professional obligation I need to act on more directly?

It is also worth noting that MI skills and the spirit retain value, even in consultations that are not primarily oriented toward long-term behaviour change. Reflective listening, summarising, and supporting autonomy can improve the quality of conversations about treatment decisions, end-of-life care, and other complex choices where the goal is not sustained behaviour change but helping a client reach a decision that is consistent with their own values and their animal’s needs [39]. Knowing when to step back from a guiding style and move toward a more directive approach is itself an ethical skill, and one that develops through the type of honest self-examination that ethical reflexivity requires.

## 7. Current Evidence, Research Gaps, and Future Priorities

In human healthcare, MI has been evaluated across thousands of studies spanning multiple clinical contexts, and meta-analytic evidence supports its effectiveness across a range of health behaviours when delivered with adequate fidelity [54,117]. The veterinary evidence base is considerably more modest, with two general overviews of MI for veterinary professionals [23,24] and 15 primary research studies. The evidence is reviewed here, organised around four questions: What does baseline veterinary communication look like? Can veterinarians be trained to communicate in a more MI-consistent manner? Does that training change what clients say and do? And what happens to the MI skills after training ends? A summary of included studies is presented in Table 1, and the section concludes by identifying the key limitations and priorities for future research.

### 7.1. Characterising Baseline Veterinary Communication Styles

Research examining baseline veterinary communication provides the starting point for evaluating MI’s potential in practice. Across different species, countries, and practice settings, there have been consistent findings that veterinary communication is predominantly directive, information-heavy, and persuasion-oriented, with limited attention given to client perspective, agenda-setting, shared decision-making, or acknowledgement of client autonomy [14,16,118].

Bard and colleagues [14] documented this pattern through qualitative analysis of UK cattle veterinarian consultations, finding a solution-focused communication style in which veterinarians dominated the agenda, relied heavily on closed questions, and made minimal effort to explore client motivations or perspectives. Svensson, Emanuelson, and colleagues [15] extended this analysis through MITI coding of both role-play and real on-farm consultations with 42 Swedish dairy cattle veterinarians, and found that giving information, questioning, and persuasion dominated across both settings, while seeking collaboration and emphasising autonomy were mostly absent. Interestingly, MI-nonadherent behaviours increased with years of veterinary experience in herd health consulting, while relational scores were lowest in veterinarians with either the most or least experience. In companion animal practice, Enlund and colleagues [118] found similarly low spontaneous use of MI-consistent behaviours among eight small animal veterinarians in simulated dental home care consultations, with cultivating change talk scoring at the minimum possible in every recording. Dorrestein and colleagues [16] observed comparable patterns in real farm health visits in Belgium, using both the Calgary-Cambridge Guide and a modified MITI for 52 recorded consultations with a focused MITI-codeable segment. Partnership and empathy were present but inconsistent, while autonomy-supportive behaviours were nearly absent, closed questions outnumbered open questions two to one, and persuasion appeared in 73% of the MITI-coded visits.

It is worth noting that most of these studies rely on role-play or simulated consultations rather than real clinical encounters, and the Bard 2023 [119] findings discussed below suggest that real consultation performance may be even further from MI-consistent communication than these baseline studies indicate.

### 7.2. Training Interventions and Communication Outcomes

Training studies in the veterinary literature vary considerably in design, duration, and outcome measurement, making direct comparisons difficult. The evidence base is dominated by role-play rather than real consultation assessments, a limitation that is itself a key finding of this literature review. Across studies, longer and more intensive training programmes produce greater skill gains, but even the most rigorous programmes have produced moderate competency in only a minority of participants. Self-report outcomes without objective fidelity assessment are common in the non-cattle literature, limiting conclusions about actual skill development. The consistent finding across studies is that brief training produces measurable but modest changes, that competency thresholds are rarely met, and that skills decay over time without ongoing practice and support.

The most rigorous training intervention in the veterinary literature is the six-month programme evaluated by Svensson and colleagues [66], involving 38 Swedish dairy cattle veterinarians across six workshops totalling 36 h, with self-directed reading and recorded practice between sessions and MITI-coded feedback throughout. All participants improved in at least one MITI parameter, and significant improvements were found in all but three of the 16 statistically evaluated variables. Perceived relevance and satisfaction with the programme were high. However, only 29% of participants reached moderate MITI competency, and veterinarians with very little or very substantial herd health consulting experience showed less improvement in cultivating change talk—a finding the authors suggest may reflect established communication habits being more resistant to change.

Bard and colleagues [37] examined the effects of a much briefer MI training programme with 14 UK cattle veterinarians trained either via two-hour sessions or a single five-hour session, using pre-post analysis of 31 real herd health consultations. Despite the brevity of the training, veterinarians showed measurable changes in communication behaviour after training, including more reflective statements, greater partnership orientation, and more emphasis on client language in favour of change goals. Sequential linguistic analysis of 3885 communication events within the 31 consultations found that following veterinarian MI-consistent behaviours, particularly affirmation, seeking collaboration, and emphasising client choice, farmers were significantly more likely to express commitment change talk. This is a clinically important finding, demonstrating that even limited training can produce observable changes in real consultations, though the small sample and brief training duration limit the conclusions that can be drawn.

A methodologically critical finding comes from Bard and colleagues [119], who compared MITI-coded simulated and real consultations from the same 36 veterinarians, half of whom recorded before MI training and half after. Veterinarians scored significantly lower on cultivating change talk, partnership, and empathy in real on-farm consultations than in role-play, and demonstrated more giving of information and questioning in real consultations, with no clear congruence between the two contexts. Despite lower absolute MI skills in real consultations, veterinarian skill ranking was consistent between contexts, and veterinarians reaching moderate skills in either context were associated with significantly more client change talk compared to untrained veterinarians. The key implication for the training literature is that studies evaluating MI competency using role-play alone, which includes most of the veterinary training research, are likely substantially overestimating the fidelity with which MI is being delivered in actual clinical practice.

The Veterinary Dialogue Trainer (VDT), an online simulation tool combining MI and Calgary-Cambridge principles, shows some potential as a low-cost supplementary training tool. Dorrestein and colleagues [120] found that 24 Finnish and 21 Swedish dairy veterinarians improved their VDT scores substantially across repeated attempts, with most reaching scores above 80% after a mean of four attempts. Whether VDT competency translates into real consultation performance remains untested, but the tool’s accessibility and scalability make it worth further investigation as a complement to workshop-based training.

Two studies have examined MI training in non-clinical contexts. Williams and colleagues [121] evaluated a bespoke five-day MI training programme for 26 equine welfare officers, finding significant increases in self-reported knowledge and confidence, with participants reporting a beneficial application of MI skills in welfare caseloads, including hoarding cases. Andrukonis and colleagues [122] evaluated a 4.5 h MI training programme for ten animal shelter employees, finding increased self-reported confidence and frequency of MI use following training. Both studies relied on self-report outcomes with no objective fidelity assessment, limiting the conclusions about actual skill development or clinical impact.

### 7.3. Effects on Client Communication and Behaviour

The most direct test of MI’s value in veterinary practice is whether more MI-consistent practitioner communication influences what clients say and do during and after the consultation. The evidence is suggestive but limited, with only four studies examining client communication or behavioural outcomes, and most with methodological limitations that prevent firm conclusions about whether MI-consistent communication produces sustained behaviour change or improvements in animal welfare.

Svensson and colleagues [123] coded on-farm consultations between 36 dairy veterinarians, half of whom had received the six-month MI training, and 170 farm visits, assessing client verbal responses using the CLEAR coding system. Veterinarians’ MI skills were associated with client change talk, with clients of veterinarians reaching moderate proficiency, expressing 1.6 times more change talk than clients of untrained veterinarians. This is consistent with the technical hypothesis that MI-consistent communication elicits change talk, which, in turn, predicts behaviour change. Results regarding sustain talk and the proportion of change talk were inconclusive, and the moderate proficiency threshold used has not been validated against behaviour change outcomes in veterinary contexts.

Baker and colleagues [124] provide the most direct evidence of MI-associated behaviour change in a veterinary-adjacent context. Using an MI-facilitated action-planning approach with 29 laying hen farmers, a trained facilitator used open questions, affirmations, reflective listening, and structured interviews to co-develop bespoke feather cover action plans with each farmer. Of the 26 farmers available at follow-up, 80% made management changes, with 90% of free-range farmers making changes and 50% of those using enriched cages. At nine-month follow-up, 67% of planned changes in free-range flocks had been achieved. However, the absence of a control group means the contribution of MI specifically cannot be isolated, and no formal fidelity tools were used to assess adherence to MI during the conversations, thus limiting the conclusions that can be drawn about what trained veterinarians might achieve.

The most rigorous behavioural outcome study in the veterinary literature is the three-year randomised controlled trial by Enlund and colleagues [125], comparing MI with traditional advice and a control group of dog owners receiving telephone consultations about dental home care. Tooth brushing frequency was significantly higher in the MI group than in the control group, and the plaque index was significantly lower. However, there were no significant differences between the MI and traditional advice groups on most measures. Satisfaction scores were similar across groups. The MI group showed more MI-adherent behaviour, but the same rate of nonadherent behaviours, and it is unclear as to whether the overall MITI competency threshold was met, raising questions about whether full MI was actually delivered. The single counsellor limits generalisability, with the authors noting that the counsellor’s experience in dental home care advice may have produced effective communication across both intervention conditions.

Svensson and colleagues [84] examined whether MI training was associated with improved performance in veterinary herd health management more broadly, including written health plans, implementation of recommendations, client satisfaction, and time allocated to herd health visits. No statistically significant effects were found on any of the eight performance variables, though a consistent pattern of numerically better herd performance outcomes in trained veterinarians suggested a direction of effect that the study was substantially underpowered to detect, with a post hoc calculation indicating 182 veterinarians would have been needed. Veterinarians with moderate MI skills spent 2.14 times more time on herd health visits than untrained veterinarians; however, this difference was not statistically significant, and the confidence interval was wide.

### 7.4. Implementation and Sustainability

Research on what happens after MI training ends is limited to a single qualitative study in the veterinary literature. Svensson and Bergeå [67] interviewed eight dairy cattle veterinarians who had completed the six-month training programme and seven animal welfare inspectors with varying MI training, identifying five themes influencing post-training MI use: the nature of the case and client, skill acquisition and retention, time pressures, personal factors, and level of support and resources. Only two of the eight veterinarians described using MI frequently after training, with the remainder describing more moderate use. Skills decay was linked to insufficient practice, with participants reporting that maintaining MI engagement was effortful and that reverting to directive habits was easier under stress or time pressure. Organisational support and personal fit with the MI style were identified as important enablers of sustained use. Whether these patterns generalise beyond dairy cattle practice in Sweden remains unknown, and the absence of further implementation research in veterinary contexts is itself a significant gap.

### 7.5. Limitations and Future Research Priorities

The veterinary MI literature shares many of the methodological limitations documented in human healthcare research, but it is further constrained by the concentration of studies in a single species and setting. The evidence base is dominated by production animal practice, particularly dairy cattle, with most research conducted by a small cluster of research groups in Sweden and the UK. Several publications share participants, training programmes, or datasets, meaning the effective independent evidence base is smaller than the publication count suggests.

Across most studies, the sample sizes are small and the follow-up periods are typically short. Participating veterinarians and clients are almost universally self-selected, with more motivated veterinarians and more satisfied clients systematically over-represented, likely inflating the positive findings. Trainer qualifications and training fidelity are inconsistently reported across studies, making it difficult to evaluate whether differences in outcomes reflect differences in the quality of MI delivered or differences in the populations studied. For studies examining client outcomes, objective fidelity assessment is often absent or limited, and where it is present, it is typically based on role-play rather than real consultations. As demonstrated by Bard and colleagues [119], role-play substantially overestimates the MI competency that veterinarians actually demonstrate with real clients, meaning that studies that classify veterinarians by MI skill through using role-play and then drawing conclusions about real consultation outcomes are methodologically compromised.

Outcome measures are inconsistent across studies, with change talk, behaviour change, animal health parameters, and client satisfaction all used as proxies for MI effectiveness at different points in the causal chain. The MI causal chain, from practitioner behaviour to change talk, to commitment language to behaviour change to improvements in animal welfare, has not been demonstrated in veterinary settings. The veterinary field has largely borrowed this assumption from human healthcare, where the evidence is stronger, but the structural differences that distinguish veterinary consultations, particularly the proxy motivation problem, mean that it requires direct empirical testing rather than inference. Economic evaluation of MI training investment is almost entirely absent from the literature, which limits evidence-based decision-making at the practice and system levels.

A further unanswered question is whether a single MI-informed consultation is sufficient to produce meaningful behaviour change, or whether repeated interactions across multiple visits are needed to achieve and sustain change over time. Evidence from human healthcare suggests that the picture is complex. A meta-analysis of MI in paediatric health behaviour change found that the number of sessions was not a significant moderator of outcome [126], suggesting dose alone does not determine effectiveness. However, a systematic review of MI for physical activity found that the effects diminished over longer follow-up periods, with limited evidence of benefit beyond one year [127]. In veterinary contexts, where clients typically have only limited consultation episodes per clinical problem and where behaviour change may require sustained effort over weeks or months, understanding the minimum effective dose and the timeframe over which effects can realistically be expected is both a practical and a research priority. The existing veterinary evidence base has not yet addressed this question directly, and future studies should incorporate follow-up periods that are sufficient enough to detect whether any effects of MI-informed communication are maintained beyond the immediate post-consultation period.

The most pressing research priority is establishing whether MI-informed communication actually changes client behaviour and improves animal welfare, which requires controlled trials with standardised behavioural endpoints, objective fidelity assessments in real consultations, and sufficient follow-ups. Whether the proxy motivation problem affects the efficacy of MI has yet to be studied and, as such, is a further priority. Most veterinary MI research has been conducted in production animal settings, and there is a clear need for studies in companion animal practice where the human–animal bond and the emotional dimensions of the owner relationship may create a substantially different context for behaviour change conversations. Client perceptions of MI-informed consultations are also largely unexplored, with little attention given to how clients experience MI-consistent communication and whether their perceptions influence longer-term behaviour change.

Other priorities include studies of the effects of MI training on veterinary professional wellbeing and burnout, economic evaluations of training investment at the practice and system levels [85], development and validation of veterinary-specific fidelity measures that account for the structural features of veterinary consultations, exploration of AI-assisted MITI scoring as a scalable feedback tool [73], and the integration of MI with welfare assessment frameworks, such as the Five Domains Model [128], to support more comprehensive approaches to welfare-oriented consultation.

## 8. Discussion

As highlighted by this review, MI offers veterinarians both a relational mindset and a practical toolkit to shift clinical conversations away from the directive, authoritarian communication style that currently dominates veterinary practice and undermines the behaviour change outcomes the profession is trying to achieve. The limited evidence to date suggests that even brief MI training can shift veterinarians towards a more collaborative communication style and that more MI-consistent conversations are associated with greater client change talk. Although the veterinary evidence base is not yet sufficient to draw firm conclusions about the downstream effects this has on client behaviour or animal welfare, the consistency of MI’s effects across other clinical fields provides reasonable grounds for expecting similar outcomes in veterinary contexts.

The more difficult question is how to increase veterinary engagement with MI training. While awareness of MI is growing in the veterinary profession, uptake is still relatively limited to date, with only a small number of veterinary schools implementing MI training in their curricula, and few veterinary-specific MI training opportunities for continuing professional development. Across the MI training literature, it has been consistently shown that brief, one-off workshops without ongoing coaching and feedback can produce short-term changes in self-reported confidence, but rarely do they produce meaningful long-term shifts in clinical practice. Sustainable implementation is likely to require buy-in at the practice level, with clinical champions who can provide ongoing peer feedback and help embed a culture of reflective communication practice. Broadening the scope of MI training beyond purely clinical scenarios to include everyday interpersonal challenges with family, friends, and colleagues may also help practitioners build fluency more quickly by creating more opportunities to practise their skills outside the consulting room.

Whether the full MI framework is either necessary or achievable in routine veterinary practice is also worth considering. Short consultations covering multiple clinical concerns often leave limited space for extended motivational work, and many veterinary consultations are straightforward encounters where the client has no significant ambivalence to resolve. Others require the veterinarian to steer the client toward a particular course of action because the animal’s welfare demands it. In both cases, applying the full MI framework would be neither appropriate nor efficient. What may matter more in practice is whether the overall communication style is MI-consistent, characterised by genuine curiosity, respect for client autonomy, and collaborative rather than directive interaction, even when the full framework is not being followed. The concept of the shared moral agency proposed in this review offers one framework for navigating the tension between client autonomy and animal advocacy, but its practical implications for how MI is taught, assessed, and used in clinical practice remain to be worked out.

Closely related to this is the question of what outcomes MI research in veterinary settings should be measuring. Most veterinary MI studies to date have focused on practitioner communication outcomes, with relatively few exploring the long-term effects on client behaviour change or animal welfare. One key challenge is defining what a successful outcome looks like and over what timeframe it should be measured. Some behaviour changes may require multiple consultations across many months before any effect becomes visible, and following clients longitudinally to capture the subsequent effects on animal welfare may be beyond the scope and resources of many studies. Expanding the range of outcomes studied to include changes in consultation dynamics, client and practitioner satisfaction, reduced professional burnout, and practice-level financial benefits from improved compliance would provide a more complete picture of what MI-consistent communication is actually achieving and, as a result, will likely make a stronger case for integrating MI into veterinary practice. Developing agreed guidelines for designing, conducting, and reporting MI research in veterinary contexts would help standardise approaches to fidelity assessment and outcome measurement, making findings comparable across studies and settings, as well as building the cumulative evidence base that the responsible integration of MI into veterinary practice requires.

It is also important to be clear about what MI does not do. Veterinarians cannot and should not attempt to compel clients to change their behaviour, and MI is not a tool for overriding client decision-making or circumventing the requirement for informed consent. Its effectiveness depends entirely on clients engaging voluntarily with the process and finding their own reasons to act. This is consistent with both the legal and professional obligations veterinarians hold toward their clients across jurisdictions, and with MI’s own foundational commitment to client autonomy and self-determination. The goal of MI in veterinary practice is not to produce compliance but to support clients in making decisions that are genuinely their own and that serve the welfare of their animals.

## 9. Conclusions

MI has shown promise as a framework for improving veterinary client communication, and the case for investing in it is clear, even though the evidence base remains limited. Veterinarians who communicate in an MI-consistent way are more likely to evoke the client motivation that drives behaviour change, and behaviour change is ultimately what produces better outcomes for animals. However, getting there will take more than individual practitioners attending workshops. Veterinary schools, practices, and researchers each have a role in building the training infrastructure, organisational conditions, and evidence base that sustained implementation requires. The frameworks proposed in this review, including the three-level consultation model and the concept of shared moral agency, offer practical tools for both clinicians and researchers working toward that goal.

## Figures and Tables

**Table 1:** Summary of published studies examining motivational interviewing in veterinary practice, organised by primary outcome focus.

Study	Country	Species and/or Context	Study Design	Number of Participants (N)	Training Details	Fidelity Measure	Key Outcomes	Limitations
***Characterising baseline veterinary communication styles***								
Bard et al., 2017 [14]	UK	Dairy cattle veterinarians (lameness and mastitis)	Qualitative analysis of role-play interactions with a trained actor.	N = 15 vets, interactions with one interaction per vet lasting 11 min on average.	None (baseline characterisation).	None.	Predominantly directive, persuasive, solution-focused communication with minimal exploration of client motivation or perspective. Closed questions asked four times more frequently than open questions.	Role-play only; single actor, not real clients; small sample.
Svensson, Emanuelson et al., 2019 [15]	Sweden	Dairy cattle veterinarians (general farm scenarios)	Observational study of recorded role-play conversations with 3 trained actors and recording of on-farm consultations coded with MITI.	N = 42 vets (role-play) with 123 recorded conversations. N = 18 vets (on-farm) with 86 recorded conversations.	None (baseline characterisation).	MITI 4.2.1; 20 min per conversation coded by trained/professional coders at coding lab.	Dominant behaviours were giving information, asking questions, and persuasion. Seeking collaboration and emphasising autonomy were nearly absent. Only 12% reached fair Relational competency in role-play; none on farm. MI-nonadherent behaviours increased with years of veterinary experience. Relational scores were lowest amongst the least and most experienced vets.	Role-play and on-farm samples not matched; cross-sectional.
Enlund et al., 2021 [118]	Sweden	Small animal veterinarians (dental home care)	Observational study of simulated telephone consultations with 1 trained actor coded with MITI.	N = 8 vets with one interaction per vet lasting around 20 min on average.	None (baseline characterisation)	MITI 4.2.1; 20 min per conversation coded by trained/professional coders at coding lab.	Cultivating change talk scored at a minimum (1/5) in all 8 conversations. Emphasising autonomy was absent in all conversations. Dominant behaviours were giving information, persuasion, and asking questions. Minimal seeking collaboration and reflective listening. Paternalistic style with veterinarians dominating the conversation and making minimal attempts to involve the client.	Very small sample; single clinical scenario; single actor, not real clients.
Dorrestein et al., 2025 [16]	Belgium	Dairy cattle veterinarians (general farm scenarios)	Observational study of audio-recorded farm visits coded with CCG and modified MITI.	N = 27 vets with 127 recorded conversations from farm visits; Only 52 of the 116 suitable recordings had a segment meeting MITI coding criteria.	None (baseline characterisation).	Modified MITI 4.2.1 + CCG; recordings were rated by 6 trained final-year veterinary students.	Partnership and empathy were inconsistent; affirmation, seeking collaboration, and autonomy support were nearly absent; closed questions outnumbered open 2:1; persuasion was present in 73% of visits.	Many recordings were excluded for not meeting criteria; modified MITI limits direct comparison with other studies; and were coded by trained students rather than professional coders.
***Training interventions and communication outcomes***								
Svensson et al., 2020a [66]	Sweden	Dairy cattle veterinarians (herd health management)	Pre-post intervention study of role-play consultations coded with MITI before and after MI training.	N = 38 vets enrolled, with N =31 with complete pre- and post-role-play data, providing 92 pre-training and 91 post-training recordings.	Training consisted of six workshops totalling 36 h delivered over six to seven months, with self-directed reading, recorded practice consultations, and MITI-coded coaching and feedback between sessions. Specialist MI trainers used.	MITI 4.2.1 coded by trained/professional coders at the coding lab.	All participants improved in at least one MITI parameter. Significant improvements in 13 of 16 evaluated variables. Only 29% reached moderate competency, and 6% reached fair competency across all four summary measures. Vets with very little or very substantial herd health consulting experience improved less in cultivating change talk. High perceived relevance and satisfaction with training.	Role-play only; self-selected volunteers interested in communication training likely inflate results; competency gains not evaluated in real consultations; trainers not specifically experienced in veterinary contexts.
Bard et al., 2022 [37]	UK	Dairy cattle veterinarians (herd health consultations)	Pre-post intervention study comparing real herd health consultations before and after brief MI training, with MITI coding of veterinarian communication and CLAMI coding of farmer verbal responses.	N =14 vets providing 31 consultations (15 pre-training, 16 post-training) from 60 total trained veterinarians; only veterinarians who completed both pre- and post-recordings were included.	Brief MI training (BMIT) of approximately 4–5 h contact time, delivered either as two-hour clinical club sessions or one five-hour day session; designed and delivered by a MINT-member trainer; content included MI spirit, OARS skills, four processes, change talk, and sustain talk recognition.	MITI 4.2.1 for veterinarian communication; CLAMI for farmer verbal responses; both coded by a single coder (first author) who was blinded to pre/post status.	Significant post-training increase in reflections, reflections per question, and relational and technical summary measures. Significant decrease in MI-nonadherent behaviours. Proportion reaching fair MITI competency on relational and technical measures increased from 7% to 57%. Farmer change talk, and the proportion of consultation speech significantly increased post-training, with commitment change talk significantly more likely following MI-adherent behaviours than by chance.	Very small sample; only 14 of 60 trained veterinarians submitted recordings, suggesting self-selection bias toward motivated participants; single coder rather than professional coding laboratory; brief training duration insufficient for full MI competency; no long-term follow-up.
Bard et al., 2023 [119]	Sweden	Dairy cattle veterinarians (herd health advisory) and clients	Comparative study of MITI coding of simulated interactions and real-life on-farm consultations from the same 36 veterinarians; half recorded before MI training (cohort A, N = 18) and half after (cohort B, N = 18).	N = 36 vets; 106 simulated interaction recordings and 170 real-life consultation recordings. N = 170 farm clients with CLEAR-coded verbal responses.	Cohort A: recorded before MI training; Cohort B: recorded after MI training. same six-month training programme as Svensson 2020a. [66] Both cohorts received training at no cost. Specialist MI trainers used.	MITI 4.2.1 for veterinary coding + CLEAR coding system for client verbal responses in real-life consultations only; both coded by trained/professional coders at the coding lab.	No clear congruence between simulated and real consultation MI skills. Vets scored significantly lower in cultivating change talk, partnership, and empathy in real consultations than in role-play. More questions and giving information in real consultations.	Simulated interactions conducted by phone while real-life consultations were in person, introducing communication medium as a potential confounding variable; 33 of 36 veterinarians were female, limiting generalisability; small sample size.
Dorrestein et al., 2023 [120]	Finland and Sweden	Dairy cattle veterinarians (general scenarios)	Pilot evaluation of the online Veterinary Dialogue Trainer (VDT) simulation tool used as part of facilitated communication training events.	N = 45 vets (24 from Finland and 21 from Sweden).	VDT was used as part of facilitated training based on MI methodology and Calgary-Cambridge guidelines; Finnish participants received 2-day training with five VDT scenarios of increasing difficulty; Swedish participants received 4 h training covering the first VDT scenario only. Participants completed at least two attempts per scenario for comparison.	VDT scoring system based on MI and CCG principles across three parameters: clarifying needs, relationship building, and showing added value; scored 0–100%.	87% of participants scored below 50% on the first attempt. Finnish participants increased their mean total score from 15% to 77% between first and best attempt; Swedish participants from 40% to 87%. Majority (73%) reached above 80% after a mean of 4.0 (Finnish) or 2.8 (Swedish) attempts. Net Promoter Scores of +89, +88, and +83 indicate high participant satisfaction.	VDT score not validated against MITI; unclear whether VDT competency transfers to real practice; predetermined written responses do not replicate natural conversation; Finnish and Swedish groups received different training durations, limiting direct comparison.
Williams et al., 2022 [121]	UK	Equine welfare officers (hoarding cases)	Training evaluation study with pre-post self-assessment questionnaire and analysis of qualitative follow-up responses.	N = 26, including 13 field officers and 13 other organisation staff.	Bespoke 5-day course + follow-up day; adapted from human health and social care MI training blueprint; training delivered by lead author with extensive MI training experience.	Self-assessment questionnaire (Likert) based on Kirkpatrick evaluation model; qualitative post-course evaluation; no MITI or behavioural coding; no objective assessment of actual MI skill acquisition.	Statistically significant improvements in self-reported knowledge and confidence across all key learning objectives. Field officers reported MI made difficult welfare conversations easier and more collaborative, with some owners more willing to reduce animal numbers or accept assistance.	Self-reported outcomes only; no objective fidelity assessment; mixed participant group not exclusively field officers; non-clinical welfare context.
Andrukonis et al., 2024 [122]	USA	Animal control officers	Training evaluation study with participant surveys at five time points across 10 weeks, including one pre-training baseline survey.	N = 10 animal control officers recruited, with only 5 participating in training and 6 providing survey data.	Single 4.5 h training session delivered in hybrid format; facilitated by a National Certified Counsellor with MINT membership and two additional on-site facilitators with MI and animal welfare expertise.	12-point knowledge exam post-training; no MITI or behavioural coding; no objective assessment of actual MI skill acquisition.	Attending the training significantly predicted increased perceived opportunities to use MI, perceived ability to use MI, and self-reported frequency of MI use. Median post-training exam score was 8.5 out of 12. Neither exam score nor confidence ruler score predicted any of the three outcome measures. High participant attrition, with only one participant completing all five surveys.	Very small sample; self-reported outcomes only; no objective fidelity measure; single training session unlikely to produce sustained skill acquisition; non-clinical animal control context limits generalisability to veterinary practice.
***Effects on client communication and behaviour***								
Svensson et al., 2020b [123]	Sweden	Dairy and beef cattle veterinarians (herd health management)	Quasi-experimental study comparing MITI-coded role-play consultations and CLEAR-coded real on-farm consultations between MI-trained and untrained veterinarians.	N = 36 vets (18 MI-trained, 18 untrained controls); 170 on-farm consultations across 164 dairy and 6 beef farms.	MI group: same six-month training programme as Svensson 2020a [66]. Control group: no training; received training after study recordings were completed.	MITI 4.2.1 applied to role-play consultations to categorise veterinarians into skill groups; the CLEAR coding system applied to real on-farm consultations to assess client verbal responses.	Veterinarian MI skills associated with client change talk. Clients of veterinarians reaching moderate MI proficiency expressed 1.6 times more change talk than clients of untrained veterinarians. Sustain talk and proportion of change talk results inconclusive. None of the untrained veterinarians reached near-moderate MI skills.	Exploratory design; self-selected veterinarians and convenience sample of clients limit generalisability; MITI competency thresholds not validated for veterinary contexts; change talk not yet demonstrated to predict actual behaviour change in veterinary settings.
Baker et al., 2020 [124]	UK	Laying hens farmers	Interventional study using MI-facilitated action planning visits with follow-up assessment of behaviour change; two farm visits per farmer.	N = 29 farmers at first visit; N = 26 at follow-up (3 unavailable).	None (interviews delivered by trained MI facilitator).	Not reported (facilitator experienced in MI; no formal fidelity tool).	80% of farmers who followed up (21/26) made management changes: 90% of free-range farmers made changes; 50% of enriched cage farmers made changes. 67.8% of planned changes in free-range flocks were achieved at an average of nine months of follow-up. Farmers with higher initial engagement and motivation implemented more planned actions.	No control group; cannot attribute outcomes to MI specifically; no objective fidelity assessment; facilitator qualifications not formally described.
Enlund et al., 2024 [125]	Sweden	Dog owners (dental care advice)	Three-arm RCT comparing MI, traditional advice, and a no-contact control group; longitudinal follow-up over three years with annual telephone consultations for intervention groups and dental health assessment at study conclusion.	N = 75 dog owners (25 per group) at recruitment; 37 of 69 remaining owners completed the final dental examination (51% dropout).	All consultations were delivered by a single veterinarian (first author) specialising in veterinary dentistry, trained in MI but with limited practical MI experience; the traditional advice group received semi-standardised one-way information; the control group received no telephone consultations.	MITI 4.2.1 applied to all first conversations in both intervention groups and coded trained/professional coders in the coding lab; + some conversations were shorter than the recommended 20 min coding window.	Tooth brushing frequency was significantly higher in the MI group vs. control. Plaque index was significantly lower in the MI group vs. control. Calculus index significantly lower in the traditional advice group vs. control. No significant differences between MI and traditional advice groups on any dental health parameter. Satisfaction scores high in both intervention groups.	Single counsellor with limited MI experience limits generalisability; same person administered intervention and assessed outcomes; many conversations shorter than 20 min MITI coding window; high dropout at follow-up examination; small breed dogs from two Stockholm clinics limit generalisability; unclear whether overall MITI competency threshold was met.
Svensson et al., 2022 [84]	Sweden	Dairy cattle veterinarians (herd health management)	Quasi-experimental study comparing herd health management performance outcomes between MI-trained and untrained veterinarians across real farm visits with follow-up assessment of implementation.	N = 36 vets (18 MI-trained, 18 untrained controls); 170 cattle farm visits.	MI group: same six-month training programme as Svensson 2020a [66]. Control group: no training; received training after study recordings were completed.	MITI 4.2.1 applied to role-play consultations to categorise veterinarians into skill groups; coded by trained/professional coders at the coding lab.	No statistically significant effects of MI training on any of the eight performance variables. Trained veterinarians showed a consistent pattern of numerically better outcomes than untrained veterinarians across most variables, though differences were small and non-significant. Veterinarians with moderate MI skills spent 2.14 times more time on visits than untrained veterinarians, but the confidence interval was wide and the effect non-significant. Median implementation of recommended measures 66.7% across all groups. Client satisfaction was high across all groups.	Substantially underpowered; post hoc calculation indicated 182 veterinarians needed to detect meaningful differences; self-selected volunteers likely overrepresent interest in herd health consulting; complex consultation outcomes difficult to attribute to communication style alone; short follow-up of three to six months.
***Implementation and sustainability after training***								
Svensson and Bergeå, 2025 [67]	Sweden	Dairy cattle veterinarians + animal welfare inspectors (AWI)	Qualitative study using semi-structured telephone interviews exploring factors influencing MI use and perceived usefulness after training.	N = 8 vets and N = 7 animal welfare inspectors; all selected on the basis of having reached moderate or near-moderate MITI competency post-training.	Veterinary participants had completed the same six-month MI training programme as Svensson 2020a [66]. AWI participants had received training of varying designs, including a longer concept equivalent to the veterinary training and a shorter digital training of approximately ten hours, plus a one-day seminar with a certified MI trainer.	Not applicable; post-training qualitative study. MITI competency was used as an inclusion criterion, but skills were not reassessed at the time of the interview.	Five themes identified: case and client characteristics, acquisition and retention of skills, time, personal factors, and level of support and resources. Only two of eight veterinarians described using MI frequently after training. AWI participants had more routinised MI skills than veterinary participants, attributed to greater organisational support and more advisory work in their role. Skills decay linked to insufficient practice, with organisational support, peer networks, and personal fit with MI style identified as important enablers of sustained use.	Small qualitative sample selected for having reached competency, likely biasing toward more positive experiences; skills not reassessed with MITI at time of interview, so actual retention cannot be confirmed; AWI participants received heterogeneous training concepts, limiting comparison with veterinary participants; retrospective recall of training experience.

AWI = animal welfare inspectors, CCG = Calgary-Cambridge Guide; CLEAR = Client Language Easy Rating; MI = motivational interviewing; MITI = Motivational Interviewing Treatment Integrity; OARS = Open questions, Affirmations, Reflective listening, Summaries; RCT = randomised controlled trial, VDT = Veterinary Dialogue Trainer. Studies are grouped by primary outcome focus.

## Data Availability

No new data were created or analysed in this study. Data sharing is not applicable to this article.

## Abbreviations

The following abbreviations are used in this manuscript:

AWI	Animal Welfare Inspector
BECCI	Behaviour Change Counselling Index
CCG	Calgary-Cambridge Guide
CLEAR	Client Language Easy Rating
COM-B	Capability, Opportunity, Motivation-Behaviour
MI	Motivational Interviewing
MINT	Motivational Interviewing Network of Trainers
MISC	Motivational Interviewing Skills Code
MITI	Motivational Interviewing Treatment Integrity Code
OARS	Open-ended Questions, Affirmations, Reflections, Summaries
SDT	Self-Determination Theory
VCPR	Veterinarian-Client-Patient Relationship
VDT	Veterinary Dialogue Trainer
